# Oral Myiasis Affecting Gingiva in a Child Patient: An Uncommon Case Report

**DOI:** 10.1155/2016/2197450

**Published:** 2016-01-03

**Authors:** Fareedi Mukram Ali, Kishor Patil, Sanjay Kar, Atulkumar A. Patil, Shabeer Ahamed

**Affiliations:** ^1^Department of Oral and Maxillofacial Surgery, SMBT Dental College and Hospital, Sangamner, Maharashtra 422608, India; ^2^Department of Oral Pathology and Microbiology, SMBT Dental College and Hospital, Sangamner, Maharashtra 422608, India; ^3^Department of Oral and Maxillofacial Surgery, Mansarovar Dental College, Hospital & Research Centre, Bhopal, Madhya Pradesh 462001, India; ^4^Department of Dentistry, Dr. Vaishampayan Memorial Government Medical College, Solapur, Maharashtra, India; ^5^Department of Periodontics, Malabar Dental College, Edapal, Kerala 679578, India

## Abstract

Certain dipteran flies larvae causing invasion of the tissues and organs of the humans or other vertebrates are called as myiasis, which feed on hosts dead or living tissues. It is well documented in the skin and hot climate regions; underdeveloped countries are affected more commonly. Oral cavity is affected rarely and it can be secondary to serious medical conditions. Poor oral hygiene, alcoholism, senility, or suppurating lesions can be associated with the oral myiasis. Inflammatory and allergic reactions are the commonest clinical manifestations of the disease. In the present case, gingiva of maxillary anterior region was affected by larval infection in a 13-year-old mentally retarded patient.

## 1. Introduction

The term myiasis is derived from the Greek word “myia,” which is used for fly and it means invasion of organs or tissues of vertebrate animals or humans by dipteral larvae. The term myiasis was coined in 1940 by* F. W. Hope*.* Zumpt* defined myiasis as the dipterous larva invading the human or other vertebrate animals and feeding on host's dead or living tissue, liquid body substances, and ingested food for certain period of time [1–3].

It is restricted to summer months in temperate zones and all year round in the tropics, as the flies which are responsible for myiasis prefer a warm and humid environment. Myiasis is less common in humans than in the vertebrate animals [4].

The ear, nose, eyes, lungs, skin, anus, and vagina are the most common sites affected in myiasis [1, 5]. The oral tissues are not permanently exposed to the external environment and thus the oral cavity rarely provides a favorable environment for the growth of the larvae [6, 7]. The predisposing factors like poor oral hygiene, presence of periodontal pockets, open bite in the anterior part, mouth breathing during sleep, ulcerative lesions, and carcinoma can be present in the patients of oral myiasis. Most of the patients are mentally retarded, senile, immunocompromised, alcoholics, and living in poor conditions [8, 9]. In the Hindu mythology, similar condition was considered in old times as the “God's” punishment to sinners [1].

The present paper describes a rare case of gingival myiasis in a 13-year-old mentally retarded patient.

## 2. Case Report

**Figure 1:**
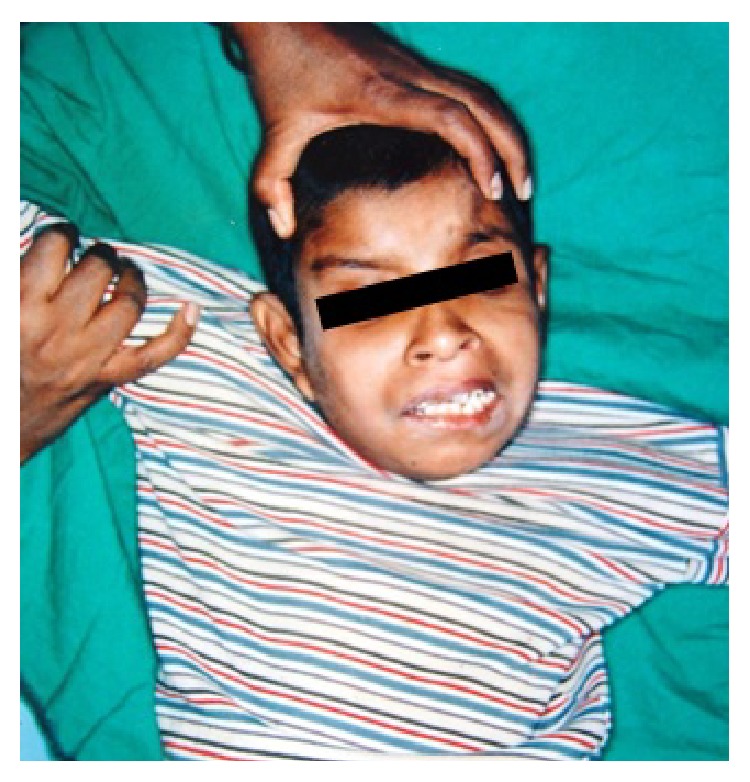
Extraoral appearance of the patient at the time of presentation.

**Figure 2:**
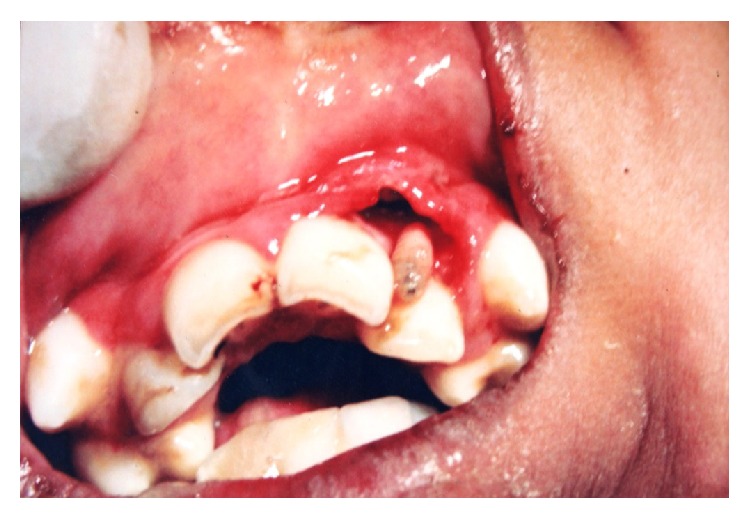
Intraoral photograph showing maggots (larvae) coming out from the maxillary anterior region after application of the turpentine oil.

A 13-year-old male patient presented to the hospital with a complaint of swelling and discomfort in maxillary anterior region since 10–12 days. On medical examination, the patient was found to be mentally retarded. The patient was from low socioeconomic background and residing in a rural area. On extra oral examination, the upper lip was swollen causing slight protrusion on the left side (Figure 1). Systemic examination of the patient was normal with normal body temperature and the regional lymph nodes were not palpable. Intraoral examination revealed an ulcerated area in the maxillary left labial vestibular region at 21 and 22. It was of 1.8 × 1.0 cm in size and a number of maggots were seen in the ulcerated area. The surrounding area of the ulcer was erythematous and swollen (Figure 2). On instrumentation, the teeth in the involved area had no mobility and his oral hygiene was poor. Based on the clinical findings, the case was provisionally diagnosed as oral myiasis. His hematological analysis was within normal limits.

### 2.1. Treatment

**Figure 3:**
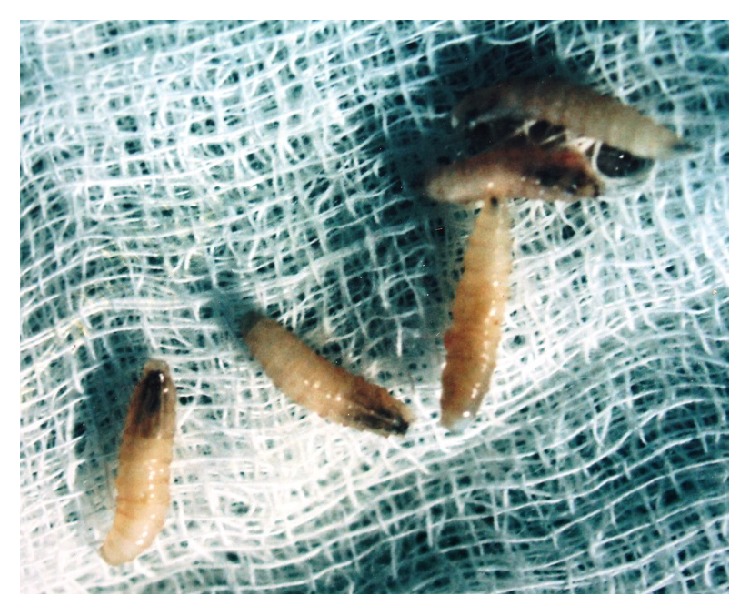
Maggots retrieved from the lesion.

The most common protocol followed for the myiasis was given for this patient. It consists of flushing affected area with turpentine oil, followed by administration of local anesthesia and removal of maggots by simple tweezers. Around 11–13 maggots were removed from the affected site (Figure 3). Ivermectin 6 mg OD for 3 days, along with metronidazole 400 mg for 5 days and analgesic, and ibuprofen with paracetamol were given to the patient. The area was then washed with saline and irrigation was done with Betadine. The procedure was repeated for 3 consecutive days until all the maggots were removed and the area was completely cleaned. On the fourth day, the site was examined for any remaining larvae and control of the infection. Then, it was sutured with 3–0 silk. Personal hygiene instructions were given to the parents of the patient.

The maggots were preserved in 10% formalin and sent to a parasitology department of medical college for identification, where they were identified as larvae of* Musca domestica* (common housefly). The larvae of the housefly were of cylindrical shape but tapering towards the head, were typical creamy white in color, and had 13 segments, of which 12 were apparent, as the first 2 were partly fused. The head was containing one pair of dark hooks.

### 2.2. Outcome and Follow-Up

After 1 month, the lesional site was found to be healed properly. On the follow-up of the patient, after 6 months, he was absolutely alright and no parasitic infestation was found in oral cavity.

## 3. Discussion

The oral myiasis, parasitic infestation of the human, is a rare condition, which mainly occurs in the rural areas. The flies of the order Diptera (maggots) are the main parasites affecting humans [1, 6]. The most common causative agent of myiasis is dipteran clade Calyptratae. It consists of four families: Calliphoridae, Sarcophagidae, Oestridae, and Muscoidea [10].

In the present case, the causative agent was identified to be of common housefly. Similar type of case reports of common housefly affecting maxillary anterior region intraorally was also reported by Bhagawati et al. [11] and Pereira et al. [1].

The life cycle of a fly consists of 4 stages: egg stage, larval stage, the pupa, and finally the adult fly. Direct inoculation into wounds and ingestion of infected materials like meat are the two ways causing infestation of the maggots in the humans. For the larval development of these flies, the intermediate host is required and the flies can lay more than 500 eggs at a time directly over the diseased tissue [3, 6, 12]. After the gravid female flies lay eggs in the tissues, the larvae hatch in about 8–10 hours, after which they invade into the surrounding tissues and cause inflammation and discomfort to the patient [1, 6, 13].

The action of proteolytic enzymes released by the surrounding bacteria causes decomposition of the tissue and helps in the feeding of the maggots [3, 14]. Larval growth causes progressive destruction and cavitation and finally forms a fibrous capsule to which they firmly adhere and cause more difficulty in dissection during surgical procedures [6, 15]. The burrowing of the larvae causes the separation of the mucoperiosteum from the bone and mild to acute pain. Thus, a patent opening is maintained with induration of the marginal tissues and raising a dome shaped “warble.” Infestation is mostly seen subcutaneously and may produce a furunculated or boil-like lesion, also called as berne [6, 15].

Larvae position themselves with their heads down to expose their posterior spiracles to the air, which makes their respiration possible. Approximately 8–10 days are required for the larvae to develop into prepupal stage after their penetration into the tissues and then they leave the host. The backward segmental hooks are useful for the anchoring of the larvae to the surrounding tissue. The larvae are photophobic; hence, they tend to hide deep into the tissues for a suitable niche to develop into pupa [1, 6].

Oral myiasis usually has male predilection because of outdoor activities and habit of neglecting oral hygiene. It is commonly seen in adults, but cases in children have also been reported. In case of oral myiasis, the most common site involved is the anterior segments of the maxillary and mandibular jaws and the palate [4, 6].

In the present case, gingiva of the maxillary anterior site was affected. Reddy et al. [8], Bhagawati et al. [11], Moshref et al. [13], Mohammadzadeh et al. [16], and Govindaraju et al. [17] also reported a case of oral myiasis affecting gingiva of the maxillary anterior region.

Clinical picture of the pulsating larvae is sufficient for the diagnosis of the oral myiasis and for the species identification it should be sent to the specialized laboratories. Mechanical removal of larvae is the most commonly used treatment [3, 6]. Local application of substances like mineral oil, ether, oil of turpentine, chloroform, mercuric chloride, ethyl chloride, creosote, phenol, saline, calomel, gentian violet, white head varnish, olive oil, and iodoform can be used for ensuring the complete removal of all larvae [6, 18, 19]. Treatment of the surrounding bacterial infection with broad-spectrum antibiotics and nutritional support of the patient with multivitamin tablets are also important. Commonly used antibiotics include ampicillin, amoxicillin, or metronidazole. Topical use of nitrofurazone and ivermectin has also been useful [3, 20, 21].

In our case, turpentine oil was applied followed by local anaesthesia and removal of the maggots by tweezer followed by antibiotic course of ivermectin, which was similar to the treatment suggested by Reddy et al. [8], Sankari and Ramakrishnan [14], Kumar and Srikumar [5], Pereira et al. [1], and Bhagawati et al. [11].

## 4. Conclusion

Oral myiasis is an uncommon condition. Parasitic infestations can be reduced by raising the quality of life and improving the personal hygiene measurements. Mental and physically disabled patients need special care to maintain oral hygiene. As dentists, it is our duty to raise awareness that a special needs patient should be exposed to proper dental intervention on regular basis as early as possible to promote cooperation and to prevent the occurrence of the disease.
